# Integrating Common Risk Factors with Polygenic Scores Improves the Prediction of Type 2 Diabetes

**DOI:** 10.3390/ijms24020984

**Published:** 2023-01-04

**Authors:** Yanina Timasheva, Zhanna Balkhiyarova, Diana Avzaletdinova, Irina Rassoleeva, Tatiana V. Morugova, Gulnaz Korytina, Inga Prokopenko, Olga Kochetova

**Affiliations:** 1 Institute of Biochemistry and Genetics, Ufa Federal Research Centre of Russian Academy of Sciences, 450054 Ufa, Russia; 2 Department of Medical Genetics and Fundamental Medicine, Bashkir State Medical University, 450008 Ufa, Russia; 3 Section of Statistical Multi-Omics, Department of Clinical & Experimental Medicine, School of Biosciences & Medicine, University of Surrey, Guildford GU2 7XH, UK; 4 Department of Endocrinology, Bashkir State Medical University, 450008 Ufa, Russia

**Keywords:** type 2 diabetes, polygenic scores, genetic predictors

## Abstract

We tested associations between 13 established genetic variants and type 2 diabetes (T2D) in 1371 study participants from the Volga-Ural region of the Eurasian continent, and evaluated the predictive ability of the model containing polygenic scores for the variants associated with T2D in our dataset, alone and in combination with other risk factors such as age and sex. Using logistic regression analysis, we found associations with T2D for the *CCL20* rs6749704 (OR = 1.68, P_FDR_ = 3.40 × 10^−5^), *CCR5* rs333 (OR = 1.99, P_FDR_ = 0.033), *ADIPOQ* rs17366743 (OR = 3.17, P_FDR_ = 2.64 × 10^−4^)*, TCF7L2* rs114758349 (OR = 1.77, P_FDR_ = 9.37 × 10^−5^), and *CCL2* rs1024611 (OR = 1.38, P_FDR_ = 0.033) polymorphisms. We showed that the most informative prognostic model included weighted polygenic scores for these five loci, and non-genetic factors such as age and sex (AUC 85.8%, 95%CI 83.7–87.8%). Compared to the model containing only non-genetic parameters, adding the polygenic score for the five T2D-associated loci showed improved net reclassification (NRI = 37.62%, 1.39 × 10^−6^). Inclusion of all 13 tested SNPs to the model with age and sex did not improve the predictive ability compared to the model containing five T2D-associated variants (NRI = −17.86, *p* = 0.093). The five variants associated with T2D in people from the Volga-Ural region are linked to inflammation (*CCR5, CCL2, CCL20*) and glucose metabolism regulation (*TCF7L, ADIPOQ2*). Further studies in independent groups of T2D patients should validate the prognostic value of the model and elucidate the molecular mechanisms of the disease development.

## 1. Introduction

The World Health Organisation defines diabetes mellitus as a metabolic disorder of multiple aetiology characterised by chronic hyperglycaemia with disturbances of carbohydrate, lipid, and protein metabolism resulting from defects in insulin secretion, insulin action, or both [1]. Type 2 diabetes (T2D) is the most common form of diabetes, accounting for 90–95% of all diabetes cases. T2D is a complex condition emerging under the influence of various genetic, behavioural, and environmental factors, with ageing and obesity being the most prominent factors contributing to its development.

Insulin resistance and persistent low-grade inflammation of the adipose tissue are important pathophysiological mechanisms of T2D progression [2]. Visceral adiposity promotes the release of adipokines (leptin, adiponectin) and adipochemokines (CCL2, CCL5, CCL17, CCL20), which are strongly implicated in insulin resistance and T2D [3]. Leptin acts through its specific receptor (LEPR), signalling satiety and suppressing appetite [4], and contributing to the regulation of glucose metabolism in an insulin-independent manner (reviewed in [5,6]). LEPR deficiency is associated with hyperglycaemia, hyperinsulinaemia, and insulin resistance [7]. Adiponectin mediates insulin sensitivity in peripheral tissues [8], and the rs17366743 variant at the *ADIPOQ* gene locus is associated with T2D [9,10]. Adiponectin also exerts its antidiabetic effect by suppressing inflammation [11]. Inflammatory cytokines including chemokines are involved in the development of T2D [12]. The results of a recent meta-analysis have demonstrated that levels of CCL2, CCL5, CCL11, and CX3CL1 chemokines were significantly higher in people with T2D than in the control participants [13]. 

In addition to leptin, other mediators play an important role in the central regulation of glucose metabolism and the development of T2D. N-methyl-D-aspartate (NMDA) receptor inhibition has been shown to increase, synchronise, and stabilise the activity of pancreatic beta cells [14]. Carbohydrate metabolism is closely interconnected with other homeostatic processes, particularly involving lipids. Triglyceride-lowering alleles in lipoprotein lipase gene (*LPL)* locus were associated with a lower risk of T2D [15]. The expression of the low-density lipoprotein receptor-related protein 5 (LRP5) gene was upregulated in a T2D model [16]. Hyperactivation of Lrp5-dependent signalling had a protective effect in hyperglycaemia and improved peripheral glucose metabolism in an insulin independent manner [17]. 

Genome-wide association studies (GWAS) have identified a number of loci associated with T2D; the most robust association was detected for polymorphisms at the transcription factor 7-like 2 (*TCF7L2*) locus [18]. Polygenic risk scores were shown to be a valuable tool for identifying the risk of many common disorders, providing a refined insight into disease pathogenesis [19]. A recent study analysing the transferability of T2D association findings between Europeans and British South Asians established the need for multi-ancestry studies to improve the characterisation of genetic contribution to the disease development, and showed that a polygenic risk score optimised for the specific population improved the identification of T2D risk [20].

In this cross-sectional study, we aimed to examine an association between common variants in genes implicated in the development of T2D, and evaluated the predictive value of polygenic scores calculated from the variants associated with T2D alone and in combination with other risk factors such as age and sex.

## 2. Results

### 2.1. Association Analysis 

We tested associations between 13 genetic variants and T2D in the entire study group (N = 1371) from the Volga-Ural region of the Eurasian continent using logistic regression analysis under the additive genetic model adjusted for age and sex. The analysis showed that five SNPs were associated with diabetes: rs1024611 at the *CCL2* gene locus, rs6749704 at *CCL20*, rs333 at *CCR5*, rs17366743 at *ADIPOQ,* and rs114758349 at *TCF7L2* (Table 1). The association of *ADIPOQ* rs17366743 and *TCF7L2* rs114758349 with T2D remained significant after additional adjustment for BMI (Supplementary Table S1), even though the analysis had lower power due to BMI data being available for only a limited number of participants (N = 589).

Polygenic score analysis assumes an additive genetic architecture with the summing of the effects of independent risk alleles [21]. We utilized the odds ratio (OR) values obtained for the genetic variants significantly associated with T2D in the regression analysis as weights for the polygenic score calculation. Additionally, we calculated both unweighted and weighted polygenic scores using all 13 genetic variants included in the study.

**Figure 1:**
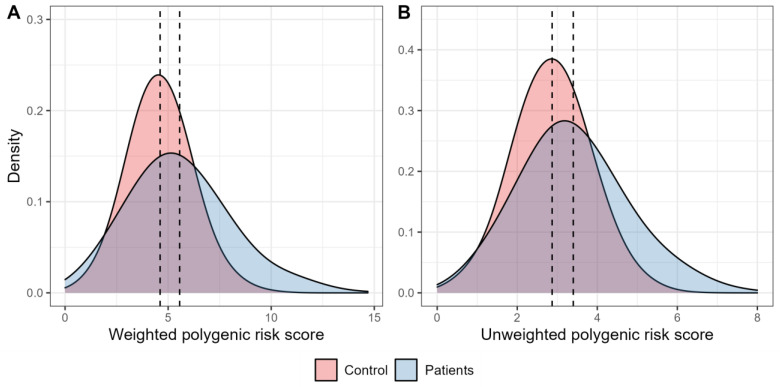
Density plots characterising the distribution of polygenic scores. (**A**). The graph characterises the distribution of density of the weighted risk scores in the control participants (red) and in people with type 2 diabetes (blue). (**B**). The graph characterises the distribution of density of the unweighted polygenic scores in the control participants (red) and in people with type 2 diabetes (blue).

Figure 1 shows the distribution of the weighted and unweighted polygenic scores in the groups of patients with T2D and healthy individuals. The mean values for both weighted and unweighted polygenic scores were higher among participants with T2D compared to the control participants (5.56 ± 0.10 vs. 4.61 ± 0.05, and 3.40 ± 0.06 vs. 2.87 ± 0.03, respectively).

The results of the analysis of the weighted polygenic scores with T2D showed that the combined effect of the five tested SNPs was associated with an increased disease risk (OR [95%CI_OR_] =1.33 [1.24–1.42], *p* = 4.56 × 10^−18^). The data obtained from the analysis of the unweighted polygenic scores also showed the association of the joint effect of these polymorphisms on the higher T2D risk (OR [95%CI_OR_] = 1.62 [1.45–1.82], *p* = 2.14 × 10^−17^).

### 2.2. Receiver Operator Characteristic (ROC) Analysis

**Figure 2:**
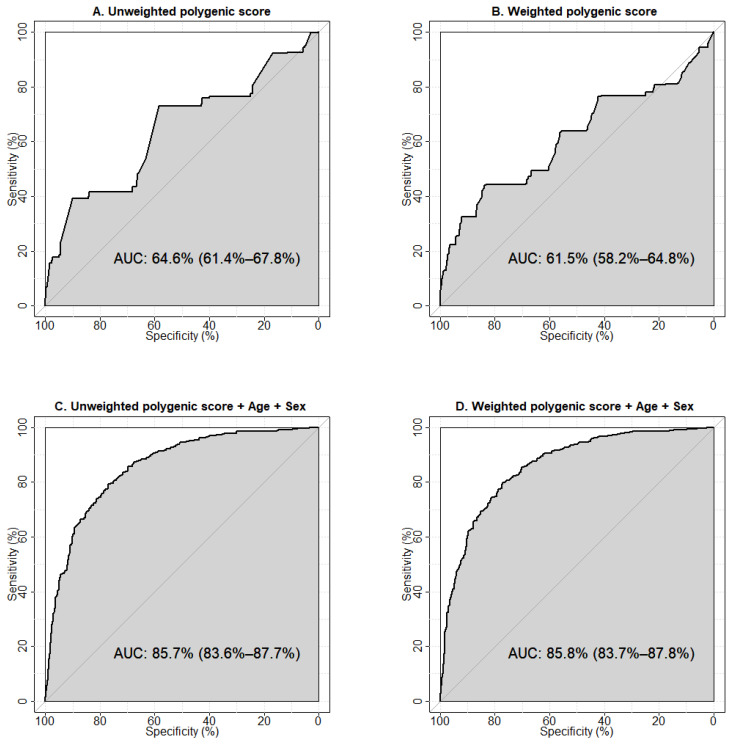
Receiver operator characteristic (ROC) curves visualising the prognostic abilities of the models to predict type 2 diabetes. (**A**) Model was constructed using the unweighted polygenic score calculated for the five genetic variants associated with type 2 diabetes in our study. (**B**) Model was constructed using the weighted polygenic score calculated for the five genetic variants associated with type 2 diabetes in our study. (**C**) Model was generated using the unweighted polygenic score for the five genetic variants in combination with age and sex. (**D**) Model was generated using the weighted polygenic score for the five variants in combination with age and sex. Sensitivity is a measure of true positive results, specificity rates true negative results. AUC (area under the ROC-curve) is a parameter used to evaluate the performance of a model (i.e., the ability to distinguish between two classes). An AUC value of 90% and higher is usually interpreted as an excellent quality of the model, 80–90% is very good, 70–80% is good, 60–70% is satisfactory, and 50–60% is unsatisfactory.

We used ROC-analysis to test the predictive abilities of the models constructed using the calculated unweighted and weighted polygenic scores and clinical parameters (age, sex, and BMI). The performances of the one-parameter prognostic models are shown in Supplementary Figure S1. The largest area under the curve (AUC) was observed for the model using age as a predictor of T2D (AUC 80.5%, 95%CI 78.2–82.9%), the smallest was for BMI (AUC 59.5%, 95%CI 54.6–64.3%). The results of the ROC analysis showed an AUC of 64.6% (95%CI 61.4–67.8%) for the unweighted (Figure 2A), and 61.5% (95%CI 58.2–64.8%) for the weighted (Figure 2B) polygenic score models constructed using five SNPs associated with T2D.

The model with unweighted polygenic scores demonstrated a greater predictive ability towards T2D. This may reflect the potential of an unweighted estimator to be more robust against errors in estimating the effect sizes arising from a limited sample size, population heterogeneity, “winner’s curse” bias, and confounding by population structure [22]. Our results indicate a moderate predictive ability of either model to correctly classify individuals with and without T2D. To evaluate the prognostic value of the predictors, we tested models including clinical factors (age + sex, age + BMI, age + sex + BMI). The largest AUC was observed for the model containing age and sex (AUC 83.8%, 95% CI 81.7–86.0%) (Supplementary Figure S2D). We then explored the prognostic abilities of models containing non-genetic factors potentially influencing T2D risk in combination with the unweighted (Supplementary Figure S3) and weighted polygenic scores (Supplementary Figure S4). We established that the model including age, sex, and weighted polygenic score demonstrated the optimal predictive ability (85.8%, 95% CI 83.7–87.8%) (Figure 2D).

Additionally, we tested the models generated using the polygenic scores calculated for all 13 genetic variants included in the study, and discovered that the model constructed using the unweighted polygenic score for 13 SNPs had a lower predictive ability than the model with unweighted polygenic score for five SNPs (AUC 59.8%, 95% CI 56.6–62.9%, vs. AUC 64.6%, 95% CI 61.4–67.8%, respectively, *p* = 4.96 × 10^−4^) (Supplementary Figure S5A,C, Supplementary Table S2). The prognostic value of the models constructed with the weighted scores calculated for five and 13 SNPs was virtually the same (AUC 61.5%, 95% CI 58.2–64.8% vs. AUC 61.5%, 95% CI 58.3–64.7%, respectively, *p* = 0.995) (Supplementary Figure S5B,D, Supplementary Table S2). Testing the models including non-genetic predictors in combination with either the unweighted (Supplementary Figure S6) or weighted polygenic score for 13 genetic variants (Supplementary Figure S7), we found that among them, the model derived from the unweighted polygenic score for 13 SNPs together with age and sex had the best predictive value (85.1%, 95% CI 83.0–87.2%) (Supplementary Figure S6D) and was lower than the optimal prognostic value for the model constructed with weighted polygenic score for five SNPs in combination with age and sex (Figure 2D), but the differences were not significant (*p* = 0.231) (Supplementary Table S2).

### 2.3. Net Reclassification Improvement Analysis

We performed net reclassification improvement (NRI) analysis using the models containing only non-genetic data (Age + Sex and Age + Sex + BMI) as the baseline, and compared them to the models with added polygenic scores for five genetic variants associated with T2D and all 13 variants included in the study (Table 2). The addition of both 5-SNP-based and 13-SNP-based weighted polygenic scores to the model constructed using age and sex improved the reclassification (total NRI 37.62% and 36.72%, NRI for cases 5.51% and 13.98%, NRI for controls 32.11% and 22.74%, respectively). The addition of the 5-SNP and 13-SNP weighted polygenic scores to the model with age, sex, and BMI showed an even greater improvement of reclassification (total NRI 41.72% and 41.50%, NRI for cases 8.70% and 16.85%, NRI for the controls of 33.02% and 24.65%, respectively). Comparing the models with the 13-SNP polygenic score to the models built with 5-SNP polygenic score, we found that inclusion of the additional variants to the models containing the non-genetic parameters did not significantly improve the reclassification (Table 2). The model with 13SNP Polygenic Score + Age + Sex compared to the 5-SNP model demonstrated a reduced overall predictive accuracy as well as worse reclassification for both the cases and controls, while the model with 13SNP Polygenic Score + Age + Sex + BMI showed better prediction for the total sample and for the controls, and worse reclassification of cases, but the results did not reach the level of statistical significance.

## 3. Discussion

In our study, we tested the associations between 13 genetic variants and T2D, and evaluated the predictive ability of the model containing polygenic scores for the five variants that were found associated with T2D in participants from the Volga-Ural region of Russia, and the model constructed using all 13 genetic variants was included in the study. We showed that the most informative prognostic model included weighted polygenic scores for the five T2D associated SNPs, and non-genetic factors such as age and sex. Previous study has shown that integrating polygenic risk scores with simple questionnaire-based risk factors including demographic, lifestyle, medication, and comorbidity data improved the risk reclassification [23]. Poly-exposure risk scores derived from the non-genetic variables were reported to identify individuals with an elevated risk of T2D [24]. The genetic structure of the population (population-specific patterns of linkage disequilibrium) could play a role in the observed effects of the studied variants on T2D differing from those previously reported, and could influence the different prognostic values of the weighted and unweighted polygenic scores. The use of multi-ethnic polygenic risk scores was demonstrated to outperform the polygenic risk scores derived in largely European populations for T2D risk prediction [25]. Inclusion of top-ranked SNPs together with gender and ethnicity affected the performance of the polygenic scores [26]. An increasing number of genetic variants for the calculation of the polygenic score could increase the predictive ability of the model [27]; however, the polygenic score derived from genome-wide T2D loci has demonstrated prognostic ability characterised by an AUC of 56%, while adding age and sex to the model has been shown to improve its predictive performance [28]. Our results indicate that expanding the polygenic score by including all of the tested SNPs did not significantly improve the predictive ability of the model (Table 2, Supplementary Table S2, Supplementary Figure S5). Replication of these findings in an independent sample is required to validate the predictive ability of the obtained model.

Among the T2D-associated signals found in our study, the rs7903146 variant at the *TCF7L2* locus is the most well-established genetic marker for T2D and the largest-effect common-variant signal for T2D in Europeans [29], first identified in 2007 in a French population [30]. The association between rs7903146 and T2D transcends ancestry and has been replicated across numerous populations including Finnish [31], Icelandic [32], Danish [33], Japanese [34], Indian [35], Punjabi Sikh [36], Mexican and Latin American [37,38], African American [39], Sub-Saharan African [40] populations as well as in multiple multi-ancestral meta-analyses [41,42,43,44]. Most recently, a meta-analysis combining the data from 122 GWAS for 180,834 individuals with T2D and 1,159,055 controls of European, East Asian, South Asian, African, African American, and Hispanic individuals with recent admixture of American, African and European ancestry [45] confirmed the association of rs7903146 with T2D. The risk of T2D conferred by the *TCF7L2* rs7903146 variant was reportedly independent from BMI and obesity [46,47]. However, testing the association between the *TCF7L2* rs7903146 variant and subtypes of T2D, determined according to various disease progression and clinical complications [48], has shown that rs7903146 was associated with severe insulin-deficient diabetes (SIDD), mild obesity-related diabetes (MOD), and mild age-related diabetes (MARD), but not with severe autoimmune diabetes (SAID) or severe insulin-resistant diabetes (SIRD) [49]. *TCF7L2* rs7903146 has been linked to insulin secretion [50], and further analysis revealed that rs7903146 overlaps with an islet enhancer and multiple islet-relevant transcription factor binding sites, and is located in islet-selective open chromatin [29,51]. In the current study, the association between *TCF7L2* rs7903146 variant and T2D remained significant after the adjustment for BMI (Supplementary Table S1), which is in agreement with previous reports that the rs7903146 variant confers T2D risk independently from BMI and obesity [46,47].

The rs17366743 SNP, a missense variant (Y111H) in exon 3 of *ADIPOQ* gene encoding adiponectin precursor, has been previously linked to an increased diabetes incidence and higher fasting glucose level in the Framingham Offspring Study [52] and has demonstrated an influence on adiponectin levels [53]. In our study, *ADIPOQ* rs17366743 showed the most pronounced effect on T2D risk, also independent from BMI (OR = 3.08, P_FDR_ = 0.013, and OR = 3.17, P_FDR_ = 2.64 × 10^−3^ with and without adjustment for BMI, respectively).

*CCR5* rs333 was previously implicated in type 1 diabetes (T1D) risk [54] and has been shown to be associated with serum levels of CCL4 [55]. CCL4 was upregulated in both people with T1D and T2D [56], and its inhibition had a protective effect on pancreatic beta-cells, increased insulin sensitivity, and delayed the progression of hyperglycaemia, suggesting the critical role of CCL4-related inflammation in the progression of diabetes [57]. *CCL2* rs1024611 is associated with gestational diabetes [58], diabetic retinopathy [59,60] and diabetic nephropathy [61]. The rs1024611 polymorphism is located in the *CCL2* enhancer region and its G allele has been associated with increased CCL2 levels in serum [62,63], cerebrospinal fluid [64], hepatic cells [65], and skin fibroblasts [66], although in a more recent GWAS, the association between rs1024611 and *CCL2* mRNA levels in serum and plasma only reached nominal significance [67]. The association between the rs6749704 variant in the promoter region of the *CCL20* gene and T2D was initially discovered in the ethnic group of Tatars [68]. This polymorphism has been linked to the immune response during HIV/AIDS infection [69], while activation of the CCL20-CCR6 axis has been discussed as a possible mechanism connecting obesity, pancreatic beta-cell inflammation, and diabetes [70].

Other studied genetic variants were not associated with T2D in our findings. It is possible that the modest sample size limited our ability to detect associations with the smaller effect variants. Another limitation of the current study was the lack of phenotypic information for some participants due to the multicentric nature of the data collection. These factors limit our ability to extrapolate the results of our study to other populations and require replication of the findings in an independent sample.

## 4. Materials and Methods

### 4.1. Study Sample

We collected information on 496 people with type 2 diabetes (T2D) (≥40 years) and 875 control participants without diabetes from April 2012 to December 2017 at Ufa City Hospital No. 21 and at the Bashkir State Medical University Clinic (Ufa, Russian Federation). The recruitment process for both the T2D and the control group has been described elsewhere [68,71]. Briefly, the inclusion criteria for the T2D group were: aged 35 years and older; T2D diagnosis established according to WHO criteria (1999–2013); lack of clinical symptoms of other types of diabetes; not related to other participants in the study. Inclusion criteria for the control group were: aged 35 years and older; absence of any clinical or laboratory symptoms of metabolic disorders; absence of a family history of diabetes; and not being related to other participants in the study. To minimise possible errors arising from population stratification, all study participants were selected from the populations historically rooted in the Volga-Ural region of the Russian Federation. Ethnic origin (up to the third generation) and the presence or absence of a family history of diabetes for all participants was established by conducting direct interviews with the potential participants.

The study was carried out in accordance with the Helsinki Declaration. The study protocol was approved by the Local Ethical Committee of Institute of Biochemistry and Genetics of Ufa Federal Research Centre of the Russian Academy of Sciences (IBG UFRC RAS), Ufa, Russia (Ufa, Protocol No. 8, 14 March 2012). All participants provided written informed consent.

### 4.2. Anthropometric Measurements and Biochemical Assays

Body weight and height were measured while participants were barefoot and wearing light indoor clothing. Body mass index (BMI; kg/m^2^) was calculated as body weight (kg) divided by height squared (m^2^). Blood samples were collected after an overnight (12 h) fast and 2 h after a meal (for the 2-h postprandial test). HbA1c was measured by high-performance liquid chromatography (ADAMS A1c HA-8182, Arkray Inc., Kyoto, Japan). Plasma glucose was measured by the glucose oxidase technique (Cobas Integra, Roche Diagnostics, Basel, Switzerland); cholesterol, triglycerides, high density lipoproteins (HDL), low density lipoproteins (LDL) were measured by photometry technique (Olympus, Hamburg, Germany). Serum C-peptide was measured by chemiluminescent immunoassays (CLIA) (human CPR CLIA Kits; IMMULITE 2000 (DPC Roche, Siemens, Los Angeles, CA, USA). The clinical characteristics of the study group are shown in Table 3.

### 4.3. Genotyping and Quality Control

Whole venous blood samples were obtained from each participant, stored at −4 °C, and used for total DNA extraction. DNA extraction and genotyping procedures were performed as previously described [68,71,72,73,74,75,76,77,78]. Genetic variants for the analysis were selected based on the previously detected associations with diabetes and related traits (Supplementary Table S3), the results of the Phenome-Wide Association Studies (PheWAS), and the variants selected for the study included those associated with metabolic traits (cholesterol levels, fat mass, T2D) and related disorders including inflammatory diseases and complications caused by T2D (Supplementary Table S4). Functional annotations of the studied genetic variants are provided in Supplementary Table S5. Allelic discrimination was performed by real-time polymerase chain reaction (PCR) with BioRad CFX96 (Bio-Rad Laboratories Inc., Hercules, CA, USA) using TaqMan SNP Genotyping Assays (Thermo Fisher Scientific, Waltham, MA, USA). As the quality control, 5% of the genotyped samples were randomly selected for re-genotyping, and all newly obtained results were identical to the previously determined genotyping data.

### 4.4. Power Analysis

Quanto software (https://pphs.usc.edu/biostatistics-software/ (accessed on 21 September 2022)) was used to calculate the statistical power of the study to detect associations with each genetic variant using the OR values established in previous studies (Supplementary Table S3), a disease prevalence of 11.6% [79] with a significance level <0.05. The obtained power estimations are provided in Supplementary Table S3.

### 4.5. Association Analysis

We tested the association between the studied loci and T2D using logistic regression analysis under the additive genetic model adjusted for age, sex, and BMI implemented in PLINK 1.9 [80]. The additive genetic model assumes that having two risk alleles has twice the impact on the outcome compared to carrying one risk allele. We applied the Benjamini–Hochberg procedure to control for the expected ratio of false positive classifications (false discovery rate—FDR) [81]. *P*_FDR_ values of less than 0.05 were considered significant.

### 4.6. Polygenic Score Calculation

Weighted and unweighted polygenic scores were calculated from the genetic variants significantly associated with T2D in the study group according to the results of a logistic regression analysis. The OR values established for these loci in the regression analysis under the additive genetic model with sex and age as covariates were used as weights for the risk alleles. If OR values detected in the initial association analysis were less than 1.0, we repeated the analysis using the alternative allele as the effect allele. The polymorphic variants located on the same chromosome were tested for linkage equilibrium.

### 4.7. Receiver Operator Characteristic Analysis

The receiver operator characteristic (ROC) analysis was applied to test the prognostic value of the obtained polygenic scores for T2D. The efficacy of the models was estimated using the area under the ROC curve (AUC), which is the measure of the ability of a classifier to distinguish between two outcomes. The models for T2D prediction were constructed using the Epi: Statistical Analysis in Epidemiology [82] and *pROC* [83] R packages.

### 4.8. Net Reclassification Analysis

We assessed the improvement in risk prediction by the models with added parameters using the net reclassification index (NRI) [84]. Continuous NRI was obtained with the nribins function implemented in the nricens: NRI for Risk Prediction Models with Time to Event and Binary Response Data R package [85]; 95% confidence intervals for NRI were calculated by bootstrapping.

## 5. Conclusions

The polygenic approach has shown its efficacy in predicting susceptibility to T2D, especially in combination with other, non-genetic risk factors such as age and sex. The five variants associated with T2D in people from the Volga-Ural region were linked to inflammation (*CCR5, CCL2, CCL20*) and glucose metabolism regulation (*TCF7L, ADIPOQ2*). The addition of other tested genetic loci previously associated with glucose metabolism and type 2 diabetes did not significantly improve the predictive ability of the model. Further studies in independent groups of T2D patients should validate the prognostic value of the model and further elucidate the molecular mechanisms of the disease development.

## Figures and Tables

**Table 1:** The results of the analysis of association between the studied genetic variants and type 2 diabetes in the study group.

Chr ^1^	Position GRCh37 ^2^	Gene	SNP ^3^	EA ^4^	MA ^5^	MAF ^6^		P_HWE_^7^	OR ^8^ (95% CI_OR_) ^9^	P ^10^	P_FDR_^11^
						Control	T2D				
1	66,036,441	*LEPR*	rs1137100	G	G	0.28	0.31	0.429	1.24 (0.89–1.72)	0.202	0.348
2	228,677,842	*CCL20*	rs6749704 *	C	C	0.28	0.39	0.094	1.68 (1.35–2.09)	2.61 × 10^−6^	3.40 × 10^−5^
3	39,307,162	*CX3CR1*	rs3732378	A	A	0.21	0.21	0.092	1.01 (0.77–1.31)	0.954	0.954
3	46,414,944	*CCR5*	rs333 *	D	D	0.06	0.10	1.000	1.99 (1.16–3.42)	0.013	0.033
3	186,572,089	*ADIPOQ*	rs17366743 *	C	C	0.03	0.09	1.000	3.17 (1.64–6.12)	6.10 × 10^−4^	2.64 × 10^−3^
8	19,819,077	*LPL*	rs320	G	G	0.24	0.26	0.294	1.21 (0.90–1.62)	0.214	0.348
10	114,758,349	*TCF7L2*	rs7903146 *	T	T	0.25	0.39	0.616	1.77 (1.37–2.29)	1.44 × 10^−5^	9.37 × 10^−5^
11	68,201,295	*LRP5*	rs3736228	T	T	0.10	0.12	0.490	0.97 (0.64–1.46)	0.874	0.947
12	14,018,777	*GRIN2B*	rs7301328	G	C	0.43	0.40	0.103	1.16 (0.95–1.41)	0.150	0.325
16	57,447,414	*CCL17*	rs223828	C	T	0.14	0.12	1.000	1.20 (0.86–1.65)	0.280	0.405
17	32,579,788	*CCL2*	rs1024611 *	A	G	0.31	0.24	0.627	1.38 (1.08–1.76)	0.011	0.033
17	32,612,402	*CCL11*	rs16969415	T	T	0.06	0.06	1.000	1.13 (0.74–1.74)	0.565	0.734
17	34,207,780	*CCL5*	rs2107538	C	T	0.25	0.25	0.261	0.95 (0.73–1.22)	0.662	0.783

^1^ Chr—chromosome, ^2^ GRCh37—Genome Reference Consortium Human Build 37; ^3^ SNP—single nucleotide polymorphism; ^4^ EA—effect allele; ^5^ MA—minor allele; ^6^ MAF—minor allele frequency; ^7^ P_HWE_—level of significance for the Hardy-Weinberg procedure; ^8^ OR—odds ratio; ^9^ 95%CI_OR_—95% confidence interval for the odds ratio; ^10^ *p*—level of significance; ^11^ P_FDR_—level of significance with the Benjamini–Hochberg adjustment. * The variants significantly associated with the type 2 diabetes genetics after the correction for multiple testing are denoted by an asterisk.

**Table 2:** The net reclassification improvement in the models with added parameters compared to the baseline.

	**Baseline (Age + Sex)**							
	**5SNP ^1^ Polygenic Score + Age + Sex**				**13SNP Polygenic Score + Age + Sex**			
	**NRI ^2^**	**SE ^3^**	**95% CI ^4^**	***p*** **-Value ^5^**	**NRI**	**SE**	**95% CI**	***p*** **-Value**
Total	37.62	7.80	19.29–49.00	1.39 × 10^−6^	36.73	7.14	22.87–50.97	2.73 × 10^−7^
Cases	5.51	5.88	−4.07–18.18	0.349	13.98	4.10	6.19–22.84	6.44 × 10^−4^
Controls	32.11	4.80	16.88–36.21	2.17 × 10^−11^	22.74	4.24	13.65–30.23	8.30 × 10^−8^
	**Baseline (Age + Sex + BMI ^6^)**							
	**5SNP Polygenic Score + Age + Sex + BMI**				**13SNP Polygenic Score + Age + Sex + BMI**			
	**NRI**	**SE**	**95% CI**	***p*** **-Value**	**NRI**	**SE**	**95% CI**	***p*** **-Value**
Total	41.72	8.70	24.67–55.62	8.44 × 10^−7^	41.50	8.95	21.21–58.79	3.53 × 10^−6^
Cases	8.70	4.65	0.26–18.37	0.061	16.85	5.10	7.61–27.15	9.58 × 10^−4^
Controls	33.02	6.03	20.76–44.44	4.27 × 10^−8^	24.65	6.28	10.44–34.96	8.72 × 10^−5^
	**Baseline (5SNP Polygenic Score + Age + Sex)**				**Baseline (5SNP Polygenic Score + Age + Sex + BMI)**			
	**13SNP Polygenic Score + Age + Sex**				**13SNP Polygenic Score + Age + Sex + BMI**			
	**NRI**	**SE**	**95% CI**	***p*** **-Value**	**NRI**	**SE**	**95% CI**	***p*** **-Value**
Total	−17.86	10.63	−37.29–3.61	0.093	4.80	16.72	−29.60–34.48	0.774
Cases	−9.74	4.95	−18.43–1.17	0.049	−2.17	7.45	−16.09–13.49	0.770
Controls	−8.11	6.81	−21.65–5.19	0.234	6.98	10.53	−16.93–24.30	0.508

^1^ SNP—single nucleotide polymorphism; ^2^ NRI—net reclassification improvement; ^3^ SE—standard error; ^4^ 95% CI—95% confidence interval; ^5^ *p*-value—level of significance; ^6^ BMI—body mass index. NRI data are given in percent.

**Table 3:** Clinical characteristics of the study group.

Characteristic	T2D ^7^, N = 496 Mean ± SD ^8^	Control, N = 875 Mean ± SD ^8^	*p* ^9^
Age (years)	55.21 ± 9.77	49.65 ± 10.9	>0.001 *
Sex: female (*N*, %)	370 (74.5)	300 (34.3)	>0.001 *
Duration of T2D (years)	7.23 ± 5.66	—	—
Age at onset (years)	54.53 ± 9.27	—	—
BMI ^1^ (kg/m^2^)	30.23 ± 5.36	28.93 ± 5.13	0.003 *
Fasting blood glucose (mmol/L)	7.35 ± 2.34	4.88 ± 0.71	>0.001 *
PPG ^2^ (mmol/L)	9.38 ± 2.62	—	—
HbA1c ^3^ (%)	7.41 ± 1.01	4.89 ± 0.60	>0.001 *
Total cholesterol	5.52 ± 1.14	5.09 ± 0.64	>0.001 *
Triglycerides	1.67 ± 1.16	1.48 ± 0.60	0.036
HDL ^4^	1.19 ± 0.50	1.09 ± 0.37	0.016
LDL ^5^	3.41 ± 4.30	2.96 ± 1.08	0.148
AC ^6^	3.76 ± 1.30	3.59 ± 0.85	0.115
C-peptide	2.16 ± 1.35	2.31 ± 0.94	0.166

^1^ BMI—body mass index; ^2^ PPG—postprandial glucose; ^3^ HbA1c—glycated haemoglobin (haemoglobin A1c); ^4^ HDL—high density lipoprotein; ^5^ LDL—low density lipoprotein; ^6^ AC—atherogenic coefficient; ^7^ T2D—type 2 diabetes ^8^ SD—standard deviation; ^9^ *p*—significance level. Statistically significant differences are denoted by an asterisk.

## Data Availability

Not applicable.

## Supplementary Materials

The following supporting information can be downloaded at: https://www.mdpi.com/article/10.3390/ijms24020984/s1. References [86,87,88,89,90,91,92,93,94,95,96] are cited in the Supplementary Material.
