# Supplementary material for: Integrating Common Risk Factors with Polygenic Scores Improves the Prediction of Type 2 Diabetes
Source: Int J Mol Sci. 2023 Jan 4;24(2):984. doi: 10.3390/ijms24020984 (PMC9866792; doi:10.3390/ijms24020984)
Supplement: Supplementary file 1 [file ijms-24-00984-s001.zip › ijms-2028364-supplementary/Description_Supplementary_Figures.pdf]

Supplementary Figure S1. Receiver operator characteristic (ROC) curves visualising the prognostic abilities of the one-parameter models to predict type 2 diabetes.

A. Model is constructed using age as predictor of type 2 diabetes. **B.** Model is constructed using sex as predictor of type 2 diabetes. **C.** Model is constructed using body mass index as predictor of type 2 diabetes. **D.** Model is generated using the unweighted polygenic score for the five variants associated with type 2 diabetes in our study as predictor of type 2 diabetes. **E.** Model is constructed using the weighted polygenic score for the five variants associated with type 2 diabetes in our study as predictor of type 2 diabetes.

Sensitivity is a measure of true positive results, specificity rates true negative results. AUC (area under the ROC-curve) is a parameter used to evaluate the performance of a model, i.e. the ability to distinguish between two classes. The AUC value of 90% and higher usually is interpreted as an excellent quality of the model, 80%-90% – very good, 70%-80% – good, 60%-70% – satisfactory, 50-60% – unsatisfactory.

Supplementary Figure S2. Receiver operator characteristic (ROC) curves visualising the prognostic abilities of the non-genetic models to predict type 2 diabetes.

A. Model is constructed using age as a predictor of type 2 diabetes. **B.** Model is constructed using sex as a predictor of type 2 diabetes. **C.** Model is constructed using body mass index as a predictor of type 2 diabetes. **D.** Model is generated using age and sex as predictors of type 2 diabetes. **E.** Model is constructed using age and body mass index as predictors of type 2 diabetes. **F.** Model is constructed using sex and body mass index as predictors of type 2 diabetes. **G.** Model is constructed using age, sex and body mass index as predictors of type 2 diabetes.

Sensitivity is a measure of true positive results, specificity rates true negative results. AUC (area under the ROC-curve) is a parameter used to evaluate the performance of a model, i.e. the ability to distinguish between two classes. The AUC value of 90% and higher usually is interpreted as an excellent quality of the model, 80%-90% – very good, 70%-80% – good, 60%-70% – satisfactory, 50-60% – unsatisfactory.

Supplementary Figure S3. Receiver operator characteristic (ROC) curves visualising the prognostic abilities of the models including the unweighted polygenic score calculated for five variants associated with type 2 diabetes to predict type 2 diabetes.

A. Model is constructed using the unweighted polygenic risk score calculated for the five genetic variants associated with type 2 diabetes in our study combined with age as predictors of type 2 diabetes. **B.** Model is constructed using the unweighted polygenic risk score calculated for the five genetic variants associated with type 2 diabetes in our study combined with sex as predictors of type 2 diabetes. **C.** Model is constructed using the unweighted polygenic risk score calculated for the five genetic variants associated with type 2 diabetes in our study combined with body mass index as predictors of type 2 diabetes. **D.** Model is constructed using the unweighted polygenic risk score calculated for the five genetic variants associated with type 2 diabetes in our study combined with age and sex as predictors of type 2 diabetes. **E.** Model is constructed using the unweighted polygenic risk score calculated for the five genetic variants associated with type 2 diabetes in our study combined with age and body mass index as predictors of type 2 diabetes. **F.** Model is constructed using the unweighted polygenic risk score calculated for the five genetic variants associated with type 2 diabetes in our study combined with sex and body mass index as predictors of type 2 diabetes. **G.** Model is constructed using the unweighted polygenic risk score calculated for the five genetic variants associated with type 2 diabetes in our study combined with age, sex and body mass index as predictors of type 2 diabetes.

Sensitivity is a measure of true positive results, specificity rates true negative results. AUC (area under the ROC-curve) is a parameter used to evaluate the performance of a model, i.e. the ability to distinguish between two classes. The AUC value of 90% and higher usually is interpreted as an excellent quality of the model, 80%-90% – very good, 70%-80% – good, 60%-70% – satisfactory, 50-60% – unsatisfactory.

Supplementary Figure S4. Receiver operator characteristic (ROC) curves visualising the prognostic abilities of the models including the weighted polygenic score calculated for five variants associated with type 2 diabetes to predict type 2 diabetes.

A. Model is constructed using the weighted polygenic risk score calculated for the five genetic variants associated with type 2 diabetes in our study combined with age as predictors of type 2 diabetes. **B.** Model is constructed using the weighted polygenic risk score calculated for the five genetic variants associated with type 2 diabetes in our study combined with sex as predictors of type 2 diabetes. **C.** Model is constructed using the weighted polygenic risk score calculated for the five genetic variants associated with type 2 diabetes in our study combined with body mass index as predictors of type 2 diabetes. **D.** Model is constructed using the weighted polygenic risk score calculated for the

five genetic variants associated with type 2 diabetes in our study combined with age and sex as predictors of type 2 diabetes. **E.** Model is constructed using the weighted polygenic risk score calculated for the five genetic variants associated with type 2 diabetes in our study combined with age and body mass index as predictors of type 2 diabetes. **F.** Model is constructed using the weighted polygenic risk score calculated for the five genetic variants associated with type 2 diabetes in our study combined with sex and body mass index as predictors of type 2 diabetes. **G.** Model is constructed using the weighted polygenic risk score calculated for the five genetic variants associated with type 2 diabetes in our study combined with age, sex and body mass index as predictors of type 2 diabetes.

Sensitivity is a measure of true positive results, specificity rates true negative results. AUC (area under the ROC-curve) is a parameter used to evaluate the performance of a model, i.e. the ability to distinguish between two classes. The AUC value of 90% and higher usually is interpreted as an excellent quality of the model, 80%-90% – very good, 70%-80% – good, 60%-70% – satisfactory, 50-60% – unsatisfactory.

Supplementary Figure S5. Receiver operator characteristic (ROC) curves visualising the prognostic abilities of the models generated for the polygenic scores calculated for 5 genetic variants associated with type 2 diabetes and models constructed for the polygenic scores calculated for 13 genetic variants included in the study.

A. Model is constructed using the unweighted polygenic risk score calculated for the 5 genetic variants associated with type 2 diabetes in our study as a predictor of type 2 diabetes. **B.** Model is constructed using the weighted polygenic risk score calculated for the 5 genetic variants associated with type 2 diabetes in our study as a predictor of type 2 diabetes. **C.** Model is constructed using the unweighted polygenic risk score calculated for 13 genetic variants included in the study as a predictor of type 2 diabetes. **D.** Model is constructed using the weighted polygenic risk score calculated for 13 genetic variants included in the study as a predictor of type 2 diabetes.

Sensitivity is a measure of true positive results, specificity rates true negative results. AUC (area under the ROC-curve) is a parameter used to evaluate the performance of a model, i.e. the ability to distinguish between two classes. The AUC value of 90% and higher usually is interpreted as an excellent quality of the model, 80%-90% – very good, 70%-80% – good, 60%-70% – satisfactory, 50-60% – unsatisfactory.

Supplementary Figure S6. Receiver operator characteristic (ROC) curves visualising the prognostic abilities of the models generated with the unweighted polygenic score calculated for 13 variants included in the study to predict type 2 diabetes.

A. Model is constructed using the unweighted polygenic risk score calculated for 13 genetic variants included in the study combined with age as predictors of type 2 diabetes. **B.** Model is constructed using the unweighted polygenic risk score calculated for 13 genetic variants included in the study combined with sex as predictors of type 2 diabetes. **C.** Model is constructed using the unweighted polygenic risk score calculated for 13 genetic variants included in the study combined with body mass index as predictors of type 2 diabetes. **D.** Model is constructed using the unweighted polygenic risk score calculated for 13 genetic variants included in the study combined with age and sex as predictors of type 2 diabetes. **E.** Model is constructed using the unweighted polygenic risk score calculated for 13 genetic variants included in the study combined with age and body mass index as predictors of type 2 diabetes. **F.** Model is constructed using the unweighted polygenic risk score calculated for 13 genetic variants included in the study combined with sex and body mass index as predictors of type 2 diabetes. **G.** Model is constructed using the unweighted polygenic risk score calculated for 13 genetic variants included in the study combined with age, sex and body mass index as predictors of type 2 diabetes.

Sensitivity is a measure of true positive results, specificity rates true negative results. AUC (area under the ROC-curve) is a parameter used to evaluate the performance of a model, i.e. the ability to distinguish between two classes. The AUC value of 90% and higher usually is interpreted as an excellent quality of the model, 80%-90% – very good, 70%-80% – good, 60%-70% – satisfactory, 50-60% – unsatisfactory.

Supplementary Figure S7. Receiver operator characteristic (ROC) curves visualising the prognostic abilities of the models generated with the weighted polygenic score calculated for 13 variants included in the study to predict type 2 diabetes.

A. Model is constructed using the weighted polygenic risk score calculated for 13 genetic variants included in the study combined with age as predictors of type 2 diabetes. **B.** Model is constructed using the weighted polygenic risk score calculated for 13 genetic variants included in the study combined with sex as predictors of type 2 diabetes. **C.** Model is constructed using the weighted polygenic risk score calculated for 13 genetic variants included in the study combined with body mass index as predictors of type 2 diabetes. **D.** Model is constructed using the weighted polygenic risk score calculated for 13 genetic variants included in the study combined with age and sex as predictors of type 2 diabetes. **E.** Model is constructed using the weighted polygenic risk score calculated for 13 genetic variants

included in the study combined with age and body mass index as predictors of type 2 diabetes. **F.** Model is constructed using the weighted polygenic risk score calculated for 13 genetic variants included in the study combined with sex and body mass index as predictors of type 2 diabetes. **G.** Model is constructed using the weighted polygenic risk score calculated for 13 genetic variants included in the study combined with age, sex and body mass index as predictors of type 2 diabetes.

Sensitivity is a measure of true positive results, specificity rates true negative results. AUC (area under the ROC-curve) is a parameter used to evaluate the performance of a model, i.e. the ability to distinguish between two classes. The AUC value of 90% and higher usually is interpreted as an excellent quality of the model, 80%-90% – very good, 70%-80% – good, 60%-70% – satisfactory, 50-60% – unsatisfactory.
