# Supplementary material for: Integrating Common Risk Factors with Polygenic Scores Improves the Prediction of Type 2 Diabetes
Source: Int J Mol Sci. 2023 Jan 4;24(2):984. doi: 10.3390/ijms24020984 (PMC9866792; doi:10.3390/ijms24020984)
Supplement: Supplementary file 1 [file ijms-24-00984-s001.zip › ijms-2028364-supplementary/Supplementary_tables_S1_S3_S4_S5.pdf]

Supplementary Table S1. The results of the analysis of association between the studied genetic variants and type 2 diabetes in the study group under additive genetic model with age, sex, and BMI as covariates

Chr[1]	Position GRCh37[2]	Gene	SNP[3]	EA[4]	MA[5]	MAF[6]		P_{HWE}[8]	OR[9] (95%CI_{OR})[10]	P[11]	P_{FDR}[12]
						Control	T2D[7]				
1	66036441	*LEPR*	rs1137100	G	G	0.29	0.309	0.312	1.23 (0.86-1.75)	0.253	0.411
2	228677842	*CCL20*	rs6749704	C	C	0.337	0.402	0.733	1.26 (0.95-1.67)	0.107	0.278
3	39307162	*CX3CR1*	rs3732378	A	A	0.171	0.212	0.806	1.11 (0.77-1.59)	0.591	0.641
3	46414944	*CCR5*	rs333	D	D	0.064	0.1	1	1.79 (0.99-3.23)	0.052	0.169
**3**	**186572089**	***ADIPOQ***	**rs17366743**	**C**	**C**	**0.032**	**0.097**	**1**	**3.08 (1.51-6.26)**	**0.002**	**0.013**
8	19819077	*LPL*	rs320	G	G	0.236	0.251	0.169	1.24 (0.9-1.71)	0.192	0.357
**10**	**114758349**	***TCF7L2***	**rs7903146**	**T**	**T**	**0.25**	**0.405**	**0.58**	**1.8 (1.35-2.39)**	**5.48×10^{-5}**	**7.13×10^{-4}**
11	68201295	*LRP5*	rs3736228	T	T	0.114	0.104	0.484	0.73 (0.47-1.15)	0.171	0.357
12	14018777	*GRIN2B*	rs7301328	G	C	0.487	0.383	0.007	1.4 (1.06-1.85)	0.017	0.073
16	57447414	*CCL17*	rs223828	C	T	0.12	0.12	0.171	1.14 (0.74-1.74)	0.555	0.641
17	32579788	*CCL2*	rs1024611	A	G	0.235	0.24	0.051	0.95 (0.67-1.33)	0.748	0.748
17	32612402	*CCL11*	rs16969415	T	T	0.051	0.066	1	1.18 (0.65-2.15)	0.585	0.641
17	34207780	*CCL5*	rs2107538	C	T	0.277	0.226	0.727	1.15 (0.82-1.62)	0.413	0.596

[1]Chr – chromosome, [2]GRCh37 – Genome Reference Consortium Human Build 37; [3]SNP – single nucleotide polymorphism; [4]EA – effect allele; [5]MA – minor allele; [6]MAF – minor allele frequency; [7]T2D - type 2 diabetes; [8]P_{HWE} – level of significance for the Hardy-Weinberg procedure; [9]OR – odds ratio; [10]95%CI_{OR} – 95% confidence interval for the odds ratio; [11]P – level of significance; [12]P_{FDR} – level of significance with the Benjamini-Hochberg adjustment. The variants significantly associated with type 2 diabetes genetic after the correction for multiple testing are shown in bold.

Supplementary Table S3. Genetic variants, included in the study, and previously detected associations with diabetes and related traits

Chr[1]	Position GRCh37[2]	Gene	SNP[3]	MA[4]	RA[5]/ NRA[6]	EAF[7]	OR[8] (CI95%)[9]	Associated trait(s)	References	Power
1	66036441	*LEPR*	rs1137100	G	G/A	0.27	1,1 (0.81-1.60)	T2D[10]	[1]	0.19
2	228677842	*CCL20*	rs6749704	C	C/T	0.23	2.77 (1.81-4.25)	T2D	[2]	0.99
3	39307162	*CX3CR1*	rs3732378	A	A/G	0.17	1.20 (NA[11])	T2D	[3]	0.42
3	46414944	*CCR5*	rs333	D	D/Wt[12]	0.11	3.4 (1.75–6.84)	T1D[13]	[4]	0.99
3	186572089	*ADIPOQ*	rs17366743	C	C/T	0.04	1.94 (1.16-3.25)	T2D	[5]	0.96
8	19819077	*LPL*	rs320	G	G/T	0.29	6.1 (4.37-8.66)	T2D (DD[14])	[6]	0.99
10	114758349	*TCF7L2*	rs7903146	T	T/C	0.32	1.40 (1.35-1.46)	T2D	[7]	0.98
11	68201295	*LRP5*	rs3736228	T	T/C	0.13	2.88 (1.78–4.77)	T1D	[8]	0.99
12	14018777	*GRIN2B*	rs7301328	C	G/C	0.36	1.24 (1.03-1.5)	T2D	[9]	0.75
16	57447414	*CCL17*	rs223828	T	T/C	0.04	1.55 (0.41 - 2.68)	T2D	[2]	0.65
17	32579788	*CCL2*	rs1024611	G	G/A	0.32	1.54 (1.16–2.04)	T2D (DN[15])	[10]	0.99
17	32612402	*CCL11*	rs16969415	T	T/C	0.05	1.50 (1.02–2.22)	T2D	[2]	0.67
17	34207780	*CCL5*	rs2107538	T	C/T	0.16	1.41 (1.07-1.88)	T1D	[11]	0.91

[1]Chr – chromosome, [2]GRCh37 – Genome Reference Consortium Human Build 37; [3]SNP – single nucleotide polymorphism; [4]MA – minor allele; [5]RA – risk allele; [6]NRA – non-risk allele; [7]EAF – effect allele frequency ;[8]OR – odds ratio; [9]95%CI_{OR} – 95% confidence interval for the odds ratio; [10]T2D – type 2 diabetes; [11]NA – not available; [12]T1D – type 1 diabetes; [13]Wt – wild type; [14]DN – diabetic dyslipidemia; [15]DN – diabetic nephropathy.

Supplementary Table S4. The results of the Phenome-Wide Association Studies (PheWAS) for the studied genetic variants

Feature	Chr[1]	Position GRCh37[2]	rsID[3]	Gene	Allele	Associated Trait	β[4]	SE(β)[5]	P[6]
Metabolic disorders	1	66036441	rs1137100	*LEPR*	G/A	Metabolic disorders	0.038	0.011	8.00×10^{-4}
	3	39307162	rs3732378	*CX3CR1*	A/G	Total cholesterol levels	1.989	0.601	9.30×10^{-4}
	8	19819077	rs320	*LPL*	G/T	Metabolic disorders	-0.060	0.012	1.51×10^{-6}
	10	114758349	rs7903146	*TCF7L2*	T/C	Type 2 diabetes (adjusted for BMI[7])	0.241	0.007	1.10×10^{-283}
	11	68201295	rs3736228	*LRP5*	T/C	Type 2 diabetes, definitions combined	0.103	0.025	3.13×10^{-5}
	12	14018777	rs7301328	*GRIN2B*	C/G	Trunk fat mass	-0.007	0.002	8.70×10^{-4}
	16	57447414	rs223828	*CCL17*	C/T	HDL cholesterol	0.039	0.004	1.70×10^{-18}
	17	34207780	rs2107538	*CCL5*	T/C	Body mass index (BMI[7])	-0.011	0.003	3.27×10^{-4}
Other diseases	2	228677842	rs6749704	*CCL20*	C/T	Atrophic disorders of skin	0.180	0.045	6.00×10^{-5}
	3	46414943	rs333	*CCR5*	T/del	Ischaemic heart disease, wide definition	0.004	0.001	3.58×10^{-3}
	3	186572089	rs17366743	*ADIPOQ*	C/T	Inflammatory disease of uterus	-0.280	0.084	8.22×10^{-4}
	17	32579788	rs1024611	*CCL2*	G/A	Stroke	0.046	0.013	5.95×10^{-4}
	17	32612402	rs16969415	*CCL11*	T/C	Plateletcrit (PCT[8])	-0.015	0.004	2.60×10^{-4}

[1]Chr – chromosome, [2]GRCh37 – Genome Reference Consortium Human Build 37; [3]rsID – polymorphism; [4]B – beta coefficient; [5]SE(β) – standard error; [6]P – level of significance; [7]BMI – body mass index; [8]PCT (plateletcrit) – the number of circulating platelets in a unit volume of blood

Supplementary Table S5. Summarized data on functional annotations of studied genetic variants using various bioinformatics tools

Gene	rsID[1]	Gene location and nucleotide change	Amino acid change	GWAS Trait	Regulatory Potential		Expression Levels (eQTL Analysis)			Epigenetic Regulation					
					Function Prediction	Regulom score	Blood/ Blood Cells	Brain	Adipose	Histone Marks	Open Chromatin	CTCF Binding	Promoter	Enhancer	DNA Methylation
LEPR	rs1137100	c.326A>G<br>4 exon	p.Lys109Arg	Waist-hip ratio (adjusted for BMI[2], female)[12]	0.061	7	+	+	+	+	-	-	-	-	-
CCL20	rs6749704	g.228677842T>C<br>Upstream Variant		NA	0.0	3a	+	+	+	+	-	-	-	-	-
CX3CR1	rs3732378	c.935C>T<br>6 exon	p.Thr280Met	Lymphocyte counts [13]	0.163	5	+	+	+	+	-	-	-	-	-
CCR5	rs333	g.8315_8346del<br>Intron Variant		CCL4 measurement [14]	0.238	5	+	-	+	+	-	-	-	-	-
ADIPOQ	rs17366743	c.331T>C<br>3 exon	p.Tyr111His	NA	NA	3a	+	+	+	+	-	-	+	-	-
LPL	rs320	g.64850T>A<br>Intron Variant		Triglyceride measurement [15]	0.0	4	+	+	+	+	-	-	-	-	-
TCF7L2	rs7903146	g.53341C>G<br>Intron Variant		Type 2 diabetes [16]	NA	5	+	+	+	+	-	-	-	-	+
LRP5	rs3736228	c.3989C>T<br>18 exon	p.Ala1330Val	Bone mineral density [17]	0.444	1f	+	+	+	+	-	-	-	-	+
GRIN2B	rs7301328	c.366C>G<br>2 exon	Pro122=	NA	0.467	7	-	+	+	+	-	-	-	-	-
CCL17	rs223828	g.57447414T>A<br>Intron Variant		NA	0.210	2b	+	+	+	+	+	+	+	-	+
CCL2	rs1024611	g.2493A>G<br>-2578A>G<br>Upstream Variant		NA	0.208	2b	+	+	+	+	+	+	+	-	-
CCL11	rs16969415	g.4716C>A<br>Upstream Variant		NA	0.0	5	+	+	+	+	+	+	-	-	-
CCL5	rs2107538	g.4598G>A<br>Upstream Variant		Blood protein levels [18]	0.0	4	+	+	+	+	+	+	+	+	+

[1]rsID – polymorphism; [2]BMI – body mass index

References:

1. Roszkowska-Gancarz, M.; Kurylowicz, A.; Polosak, J.; Mossakowska, M.; Franek, E.; Puzianowska-Kuźnicka, M. Functional polymorphisms of the leptin and leptin receptor genes are associated with longevity and with the risk of myocardial infarction and of type 2 diabetes mellitus. *Endokrynologia Polska* **2014**, *65*, 11-16, doi:10.5603/ep.2014.0002.
2. Kochetova, O.V.; Avzaletdinova, D.S.; Morugova, T.V.; Mustafina, O.E. Chemokine gene polymorphisms association with increased risk of type 2 diabetes mellitus in Tatar ethnic group, Russia. *Mol Biol Rep* **2019**, *46*, 887-896, doi:10.1007/s11033-018-4544-6.
3. Shah, R.; Hinkle, C.C.; Ferguson, J.F.; Mehta, N.N.; Li, M.; Qu, L.; Lu, Y.; Putt, M.E.; Ahima, R.S.; Reilly, M.P. Fractalkine Is a Novel Human Adipochemokine Associated With Type 2 Diabetes. *Diabetes* **2011**, *60*, 1512-1518, doi:10.2337/db10-0956.
4. Słomiński, B.; Ławrynowicz, U.; Myśliwska, J.; Ryba-Stanisławowska, M.; Skrzypkowska, M.; Myśliwiec, M.; Brandt, A. CCR5-Δ32 gene polymorphism is related to celiac disease and autoimmune thyroiditis coincidence in patients with type 1 diabetes. *Journal of Diabetes and its Complications* **2017**, *31*, 615-618, doi:https://doi.org/10.1016/j.jdiacomp.2016.10.031.
5. Hivert, M.F.; Manning, A.K.; McAteer, J.B.; Florez, J.C.; Dupuis, J.; Fox, C.S.; O'Donnell, C.J.; Cupples, L.A.; Meigs, J.B. Common variants in the adiponectin gene (ADIPOQ) associated with plasma adiponectin levels, type 2 diabetes, and diabetes-related quantitative traits: the Framingham Offspring Study. *Diabetes* **2008**, *57*, 3353-3359, doi:10.2337/db08-0700.
6. Tetik Vardarlı, A.; Harman, E.; Bozok Çetintaş, V.; Kayıkçıoğlu, M.; Vardarlı, E.; Zengi, A.; Küçükaslan, A.; Eroğlu, Z. Polymorphisms of lipid metabolism enzyme-coding genes in patients with diabetic dyslipidemia. *Anatolian journal of cardiology* **2017**, *17*, 313-321, doi:10.14744/AnatolJCardiol.2016.7142.
7. Morris, A.P.; Voight, B.F.; Teslovich, T.M.; Ferreira, T.; Segrè, A.V.; Steinthorsdottir, V.; Strawbridge, R.J.; Khan, H.; Grallert, H.; Mahajan, A.; et al. Large-scale association analysis provides insights into the genetic architecture and pathophysiology of type 2 diabetes. *Nat Genet* **2012**, *44*, 981-990, doi:10.1038/ng.2383.
8. Souza, K.S.C.d.; Ururahy, M.A.G.; Oliveira, Y.M.d.C.; Loureiro, M.B.; Silva, H.P.V.d.; Bortolin, R.H.; Luchessi, A.D.; Arrais, R.F.; Hirata, R.D.C.; Almeida, M.d.G. The low-density lipoprotein receptor-related protein 5 (LRP5) 4037C> T polymorphism: candidate for susceptibility to type 1 diabetes mellitus. *Archives of Endocrinology and Metabolism* **2018**, *62*, 480-484, doi:10.20945/2359-3997000000057.
9. Kochetova, O.V.; Avzaletdinova, D.S.; Korytina, G.F.; Morugova, T.V.; Mustafina, O.E. The association between eating behavior and polymorphisms in GRIN2B, GRIK3, GRIA1 and GRIN1 genes in people with type 2 diabetes mellitus. *Mol Biol Rep* **2020**, *47*, 2035-2046, doi:10.1007/s11033-020-05304-x.
10. Raina, P.; Matharoo, K.; Bhanwer, A.J. Monocyte chemoattractant protein-1 (MCP-1) g.-2518A>G polymorphism and susceptibility to type 2 diabetes (T2D) and end stage renal disease (ESRD) in the North-West Indian population of Punjab. *Ann Hum Biol* **2015**, *42*, 276-282, doi:10.3109/03014460.2014.941932.
11. Zhernakova, A.; Alizadeh, B.Z.; Eerligh, P.; Hanifi-Moghaddam, P.; Schloot, N.C.; Diosdado, B.; Wijmenga, C.; Roep, B.O.; Koeleman, B.P.C. Genetic variants of RANTES are associated with serum RANTES level and protection for type 1 diabetes. *Genes & Immunity* **2006**, *7*, 544-549, doi:10.1038/sj.gene.6364326.
12. Pulit, S.L.; Stoneman, C.; Morris, A.P.; Wood, A.R.; Glastonbury, C.A.; Tyrrell, J.; Yengo, L.; Ferreira, T.; Marouli, E.; Ji, Y.; et al. Meta-analysis of genome-wide association studies for body fat distribution in 694 649 individuals of European ancestry. *Hum Mol Genet* **2019**, *28*, 166-174, doi:10.1093/hmg/ddy327.
13. Vuckovic, D.; Bao, E.L.; Akbari, P.; Lareau, C.A.; Mousas, A.; Jiang, T.; Chen, M.H.; Raffield, L.M.; Tardaguila, M.; Huffman, J.E.; et al. The Polygenic and Monogenic Basis of Blood Traits and Diseases. *Cell* **2020**, *182*, 1214-1231.e1211, doi:10.1016/j.cell.2020.08.008.
14. Ahsan, M.; Ek, W.E.; Rask-Andersen, M.; Karlsson, T.; Lind-Thomsen, A.; Enroth, S.; Gyllensten, U.; Johansson, Å. The relative contribution of DNA methylation and genetic variants on protein

biomarkers for human diseases. *PLoS Genet* **2017**, *13*, e1007005, doi:10.1371/journal.pgen.1007005.

15. Harshfield, E.L.; Fauman, E.B.; Stacey, D.; Paul, D.S.; Ziemek, D.; Ong, R.M.Y.; Danesh, J.; Butterworth, A.S.; Rasheed, A.; Sattar, T.; et al. Genome-wide analysis of blood lipid metabolites in over 5000 South Asians reveals biological insights at cardiometabolic disease loci. *BMC medicine* **2021**, *19*, 232, doi:10.1186/s12916-021-02087-1.
16. Vujkovic, M.; Keaton, J.M.; Lynch, J.A.; Miller, D.R.; Zhou, J.; Tcheandjieu, C.; Huffman, J.E.; Assimes, T.L.; Lorenz, K.; Zhu, X.; et al. Discovery of 318 new risk loci for type 2 diabetes and related vascular outcomes among 1.4 million participants in a multi-ancestry meta-analysis. *Nat Genet* **2020**, *52*, 680-691, doi:10.1038/s41588-020-0637-y.
17. Richards, J.B.; Rivadeneira, F.; Inouye, M.; Pastinen, T.M.; Soranzo, N.; Wilson, S.G.; Andrew, T.; Falchi, M.; Gwilliam, R.; Ahmadi, K.R.; et al. Bone mineral density, osteoporosis, and osteoporotic fractures: a genome-wide association study. *Lancet* **2008**, *371*, 1505-1512, doi:10.1016/s0140-6736(08)60599-1.
18. Emilsson, V.; Ilkov, M.; Lamb, J.R.; Finkel, N.; Gudmundsson, E.F.; Pitts, R.; Hoover, H.; Gudmundsdottir, V.; Horman, S.R.; Aspelund, T.; et al. Co-regulatory networks of human serum proteins link genetics to disease. *Science* **2018**, *361*, 769-773, doi:10.1126/science.aaq1327.
