# Supplementary figures and images for: Integrating Common Risk Factors with Polygenic Scores Improves the Prediction of Type 2 Diabetes
Source: Int J Mol Sci. 2023 Jan 4;24(2):984. doi: 10.3390/ijms24020984 (PMC9866792; doi:10.3390/ijms24020984)

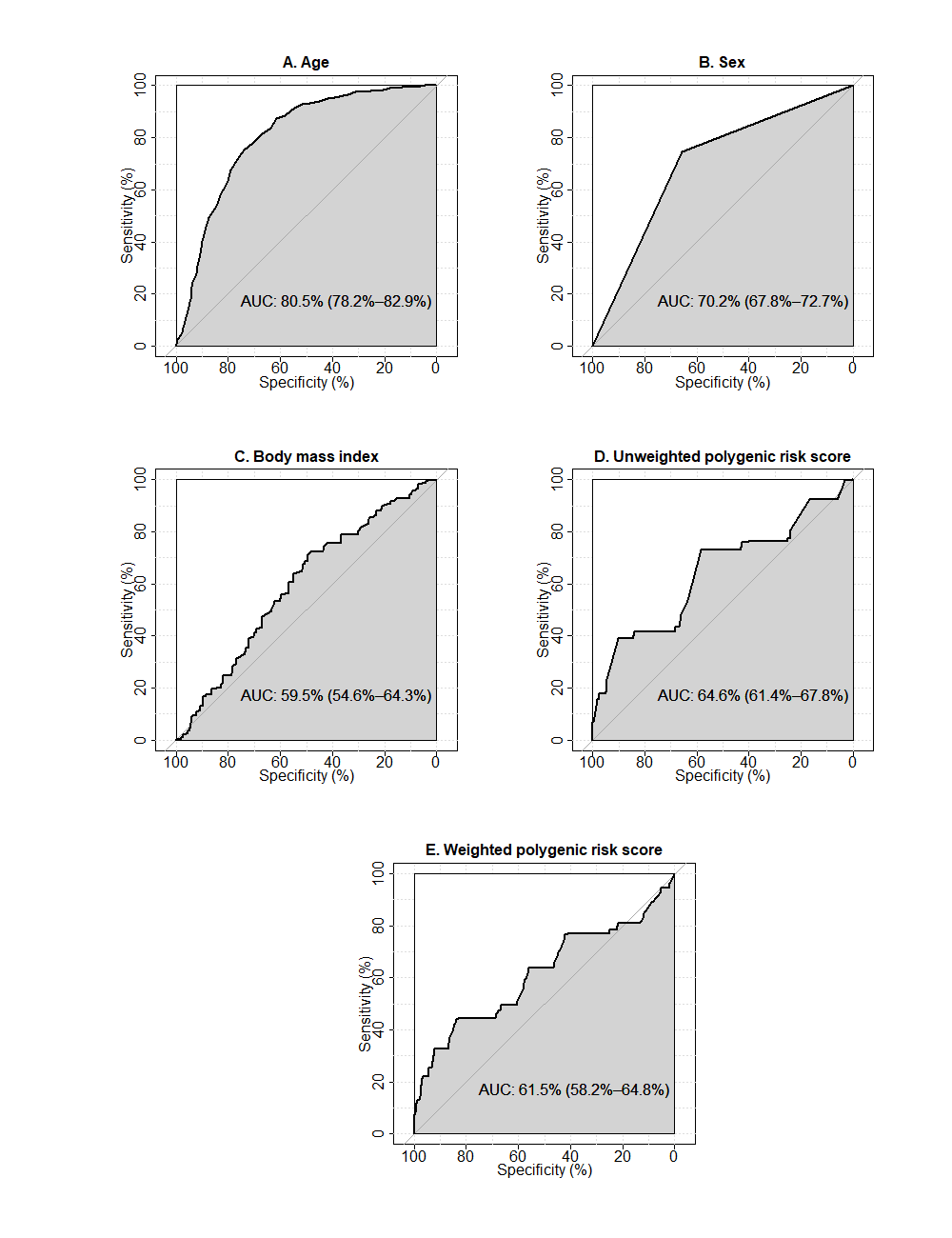

Supplement: Supplementary file 1 [file ijms-24-00984-s001.zip › ijms-2028364-supplementary/Figure_S1.png]

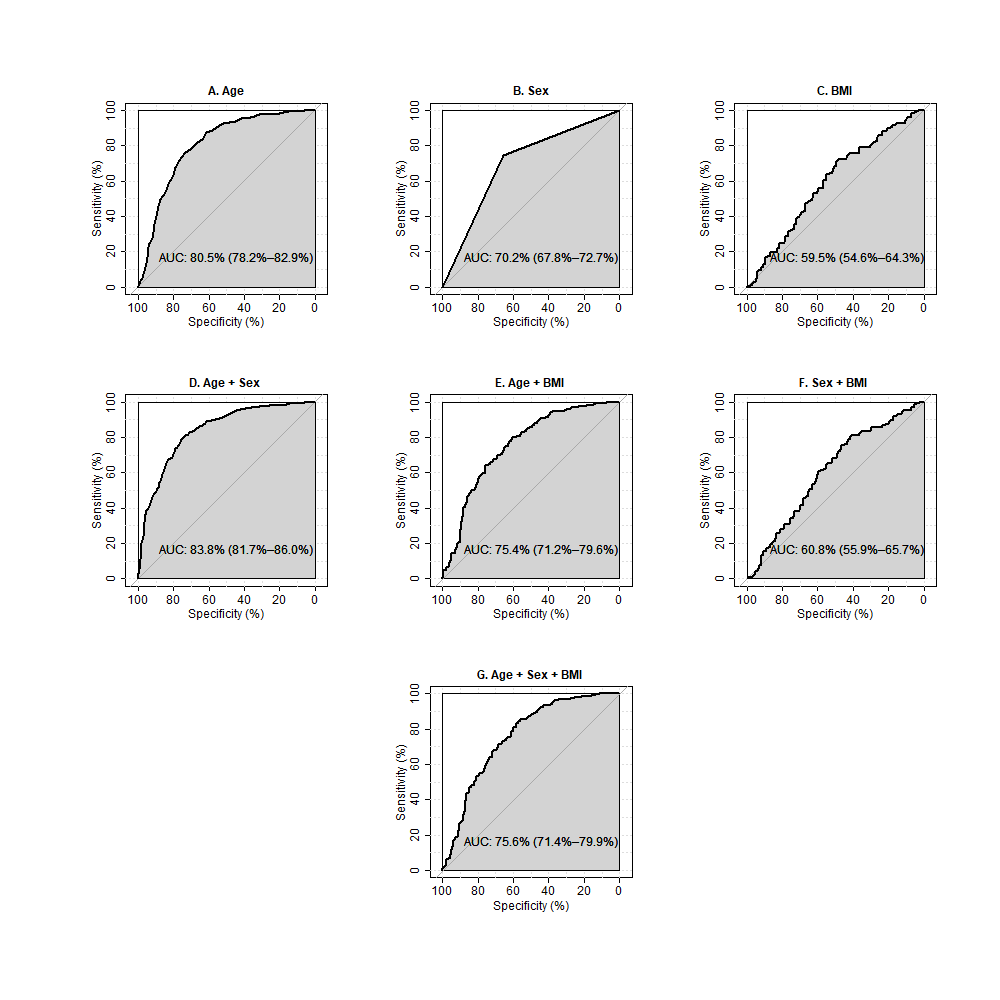

Supplement: Supplementary file 1 [file ijms-24-00984-s001.zip › ijms-2028364-supplementary/Figure_S2.png]

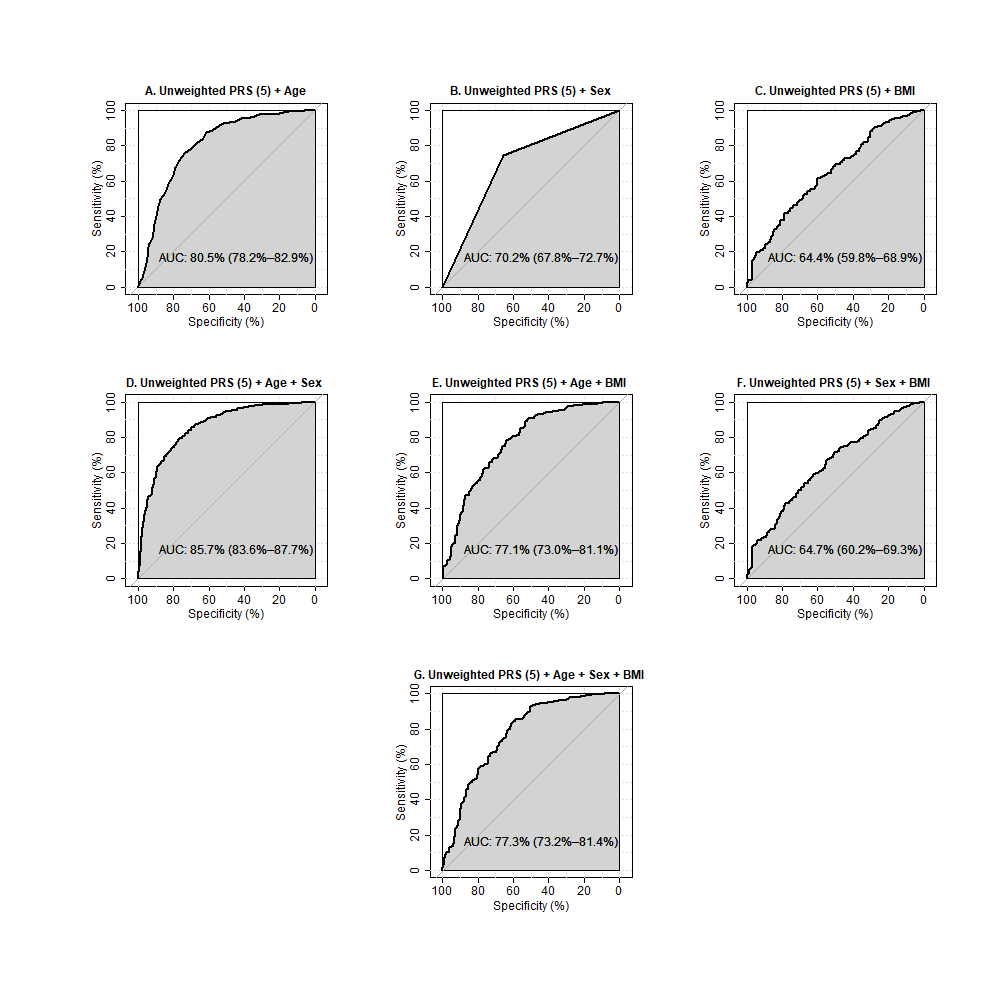

Supplement: Supplementary file 1 [file ijms-24-00984-s001.zip › ijms-2028364-supplementary/Figure_S3.png]

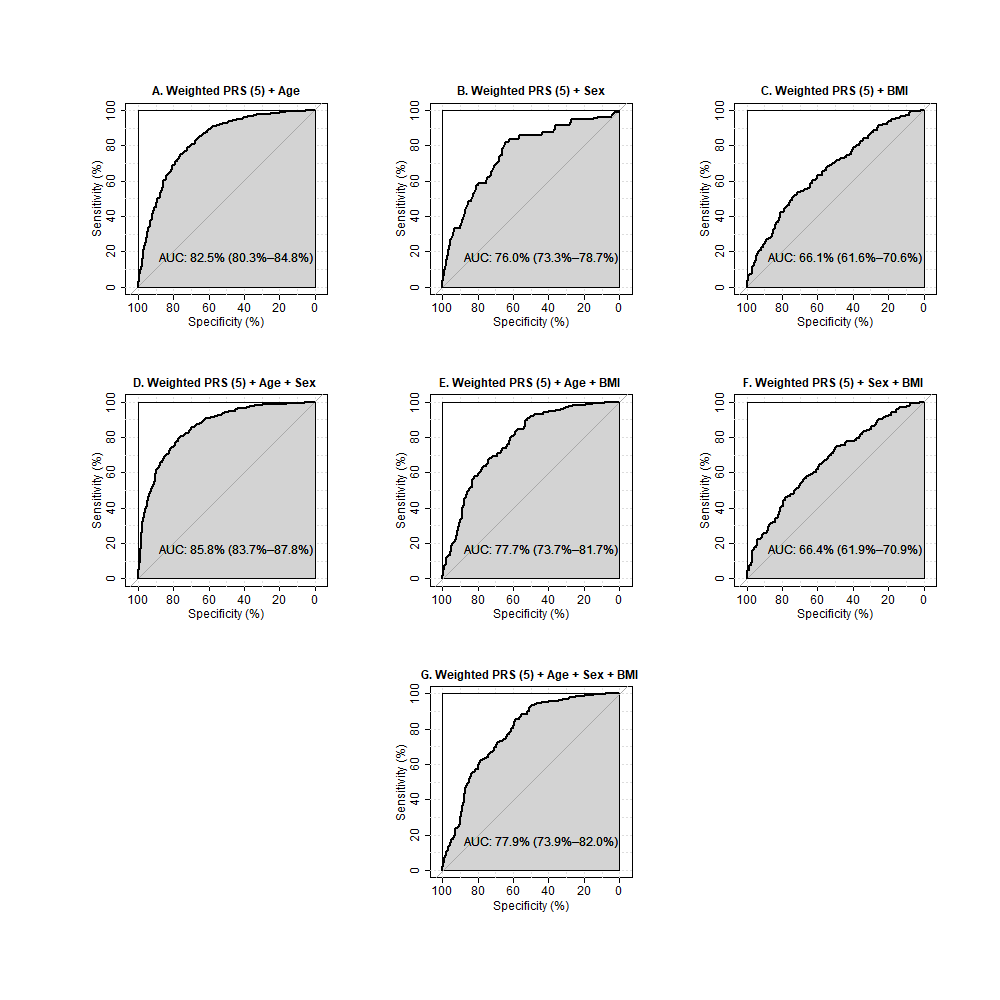

Supplement: Supplementary file 1 [file ijms-24-00984-s001.zip › ijms-2028364-supplementary/Figure_S4.png]

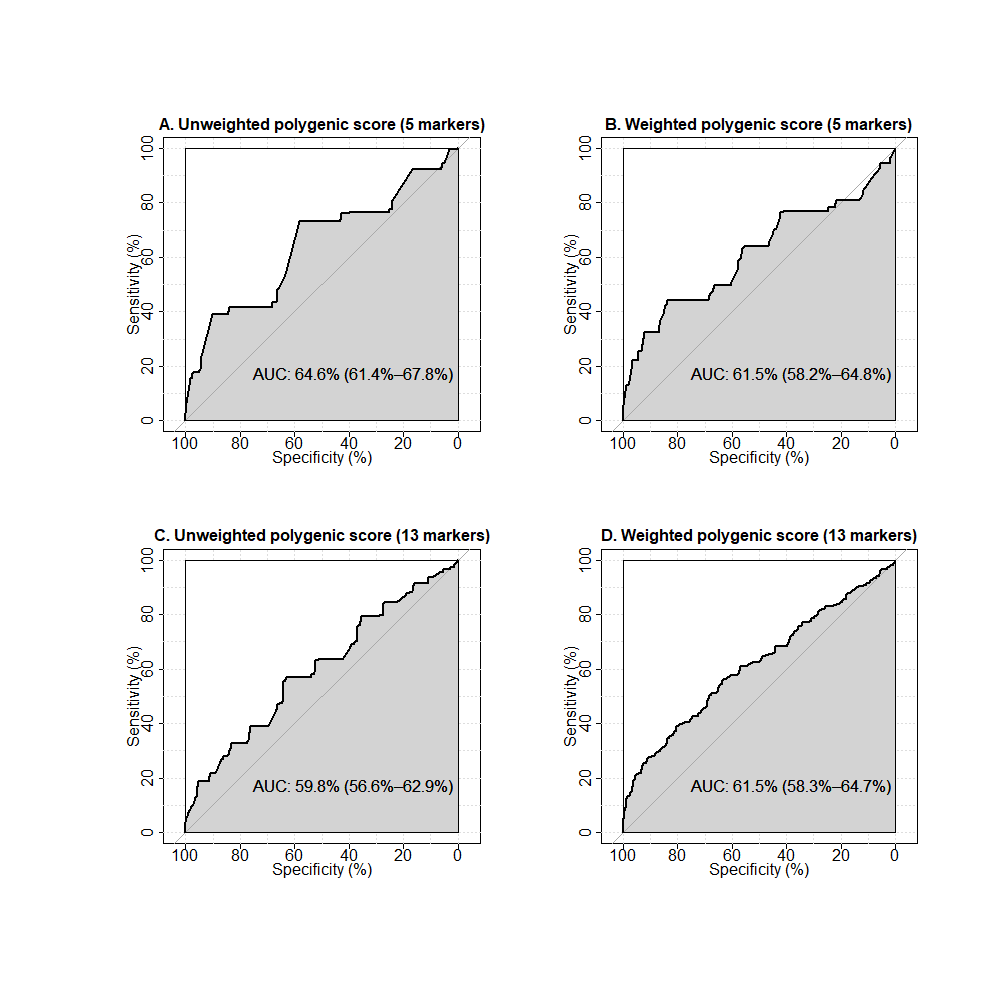

Supplement: Supplementary file 1 [file ijms-24-00984-s001.zip › ijms-2028364-supplementary/Figure_S5.png]

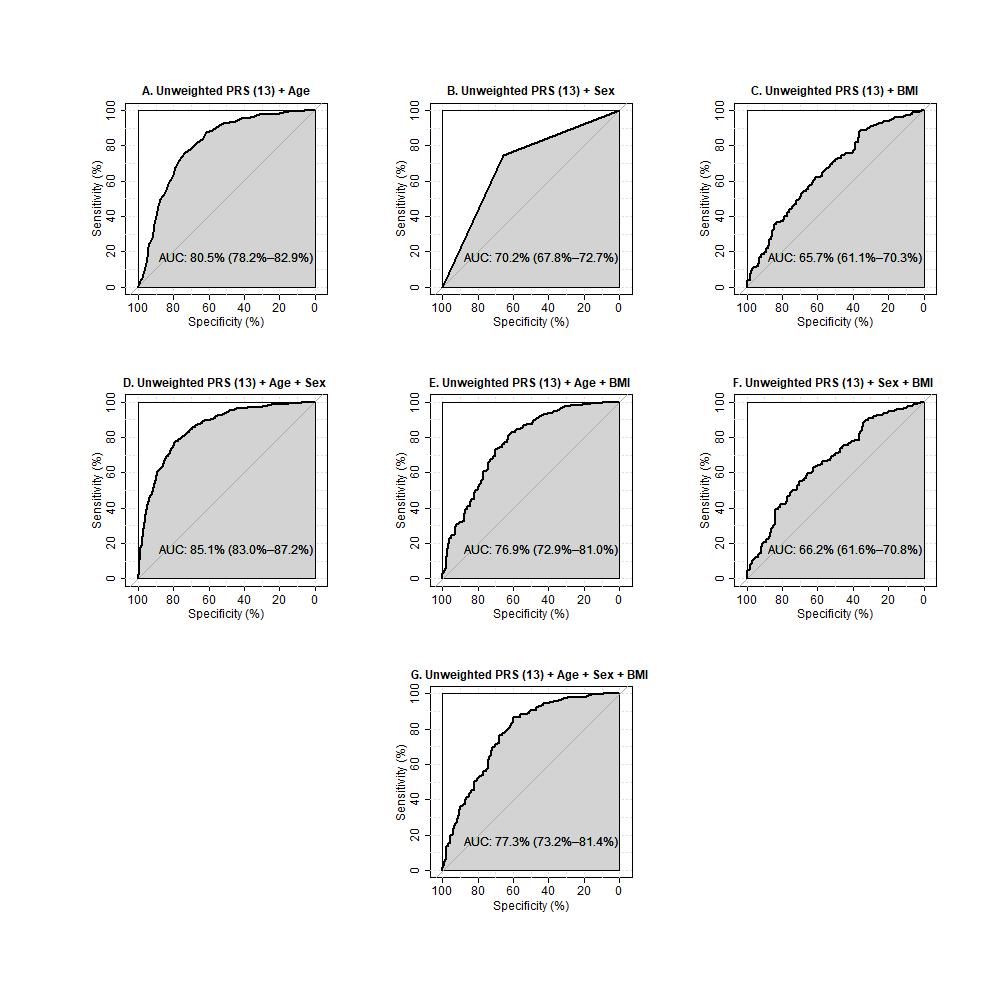

Supplement: Supplementary file 1 [file ijms-24-00984-s001.zip › ijms-2028364-supplementary/Figure_S6.png]

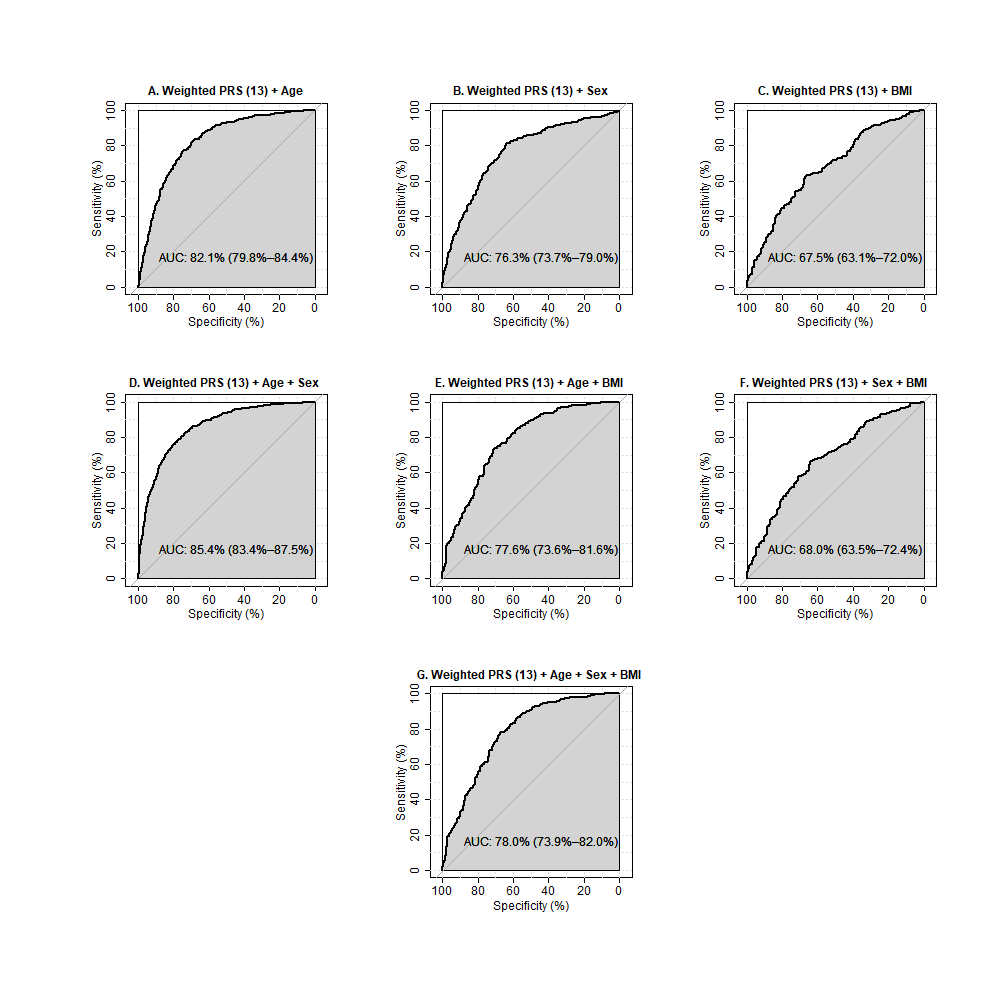

Supplement: Supplementary file 1 [file ijms-24-00984-s001.zip › ijms-2028364-supplementary/Figure_S7.png]
